# Comorbidities associated with mortality in 31,461 adults with COVID-19 in the United States: A federated electronic medical record analysis

**DOI:** 10.1371/journal.pmed.1003321

**Published:** 2020-09-10

**Authors:** Stephanie L. Harrison, Elnara Fazio-Eynullayeva, Deirdre A. Lane, Paula Underhill, Gregory Y. H. Lip

**Affiliations:** 1 Liverpool Centre for Cardiovascular Science, University of Liverpool and Liverpool Heart and Chest Hospital, Liverpool, United Kingdom; 2 TriNetX, Inc., Cambridge, Massachusetts, United States of America; 3 Aalborg Thrombosis Research Unit, Department of Clinical Medicine, Aalborg University, Aalborg, Denmark; 4 TriNetX, Inc., London, United Kingdom; Universitair Medisch Centrum Utrecht, NETHERLANDS

## Abstract

**Background:**

At the beginning of June 2020, there were nearly 7 million reported cases of coronavirus disease 2019 (COVID-19) worldwide and over 400,000 deaths in people with COVID-19. The objective of this study was to determine associations between comorbidities listed in the Charlson comorbidity index and mortality among patients in the United States with COVID-19.

**Methods and findings:**

A retrospective cohort study of adults with COVID-19 from 24 healthcare organizations in the US was conducted. The study included adults aged 18–90 years with COVID-19 coded in their electronic medical records between January 20, 2020, and May 26, 2020. Results were also stratified by age groups (<50 years, 50–69 years, or 70–90 years). A total of 31,461 patients were included. Median age was 50 years (interquartile range [IQR], 35–63) and 54.5% (*n* = 17,155) were female. The most common comorbidities listed in the Charlson comorbidity index were chronic pulmonary disease (17.5%, *n* = 5,513) and diabetes mellitus (15.0%, *n* = 4,710). Multivariate logistic regression analyses showed older age (odds ratio [OR] per year 1.06; 95% confidence interval [CI] 1.06–1.07; *p* < 0.001), male sex (OR 1.75; 95% CI 1.55–1.98; *p* < 0.001), being black or African American compared to white (OR 1.50; 95% CI 1.31–1.71; *p* < 0.001), myocardial infarction (OR 1.97; 95% CI 1.64–2.35; *p* < 0.001), congestive heart failure (OR 1.42; 95% CI 1.21–1.67; *p* < 0.001), dementia (OR 1.29; 95% CI 1.07–1.56; *p* = 0.008), chronic pulmonary disease (OR 1.24; 95% CI 1.08–1.43; *p* = 0.003), mild liver disease (OR 1.26; 95% CI 1.00–1.59; *p* = 0.046), moderate/severe liver disease (OR 2.62; 95% CI 1.53–4.47; *p* < 0.001), renal disease (OR 2.13; 95% CI 1.84–2.46; *p* < 0.001), and metastatic solid tumor (OR 1.70; 95% CI 1.19–2.43; *p* = 0.004) were associated with higher odds of mortality with COVID-19. Older age, male sex, and being black or African American (compared to being white) remained significantly associated with higher odds of death in age-stratified analyses. There were differences in which comorbidities were significantly associated with mortality between age groups. Limitations include that the data were collected from the healthcare organization electronic medical record databases and some comorbidities may be underreported and ethnicity was unknown for 24% of participants. Deaths during an inpatient or outpatient visit at the participating healthcare organizations were recorded; however, deaths occurring outside of the hospital setting are not well captured.

**Conclusions:**

Identifying patient characteristics and conditions associated with mortality with COVID-19 is important for hypothesis generating for clinical trials and to develop targeted intervention strategies.

## Abstract 2

In this analysis of electronic health records, Stephanie Harrison and colleagues investigate comorbidities and mortality in COVID-19 patients.

## Author summary

**Why was this study done?:**

- Coronavirus disease 2019 (COVID-19) has led to a public health emergency internationally.
- As of June 2020, there were over 400,000 deaths reported with COVID-19 globally and over 110,000 deaths were in the US, but many people have also recovered.
- Because of the unprecedented outbreak of COVID-19 worldwide, little is known about which underlying health conditions may impact a person’s likelihood of dying with COVID-19.
- Some previous studies have suggested being older; being from a black, Asian, or minority ethnic (BAME) background; and having certain health conditions may increase risk of death with COVID-19, but further evidence is needed to understand factors which influence this.

**What did the researchers do and find?:**

- The research utilized a network of 24 healthcare organizations in the US, which provided deidentified data from electronic medical records of patients.
- A total of 31,461 adults with COVID-19 coded in their electronic medical records were included in the study after a search of the network between January 20, 2020, and May 26, 2020.
- We determined associations between age, sex, ethnicity, comorbidities, and death with COVID-19 during the study period.
- After accounting for the other included factors in the study, being older, being male, being black or African American, and having a history of myocardial infarction, congestive heart failure, dementia, chronic pulmonary disease, mild liver disease, moderate/severe liver disease, renal disease, or metastatic solid tumor were all associated with higher odds of death with COVID-19.
- There were differences in which comorbidities were associated with death when we stratified the results by age group.

**What do these findings mean?:**

- Identifying factors associated with death with COVID-19 could help with hypothesis generating for clinical trials and identify patients who may need to be targeted for early intervention or monitoring.
- The study has limitations; for example, some health conditions may be underreported or incorrectly coded in electronic medical records at the time of data entry, and all deaths of participants may not have been captured.

## Introduction

Coronavirus disease 2019 (COVID-19) was first reported in Wuhan, China, in December 2019 [1]. Subsequently, COVID-19 was declared a Public Health Emergency of International Concern on January 30, 2020, by the World Health Organization. The first confirmed case of COVID-19 reported in the US was in Washington state on January 20, 2020 [2]. At the beginning of June 2020, 213 countries and territories had reported almost 7 million cases of COVID-19, with over 400,000 deaths reported with COVID-19 and over 110,000 deaths in the US alone [3].

An emerging evidence base has started to identify factors that associate with adverse outcomes for people with COVID-19. Older age is the most consistent risk factor for severity of COVID-19 that has emerged from the literature so far [4–6]. There is some evidence to suggest being male, black or African American ethnicity, or from certain ethnic minority backgrounds or having a history of conditions including cardiovascular or cerebrovascular diseases, hypertension, diabetes mellitus, or chronic kidney disease is associated with increased COVID-19 severity and/or mortality [5, 7–12].

More studies are needed to determine associations between comorbidities and outcomes for patients with COVID-19. Multimorbidity is closely linked to frailty status [13], which is used in decision-making for critical care admission [14]. The objective of the study was to determine associations between age, sex, ethnicity, comorbidities, and mortality of adults with COVID-19 in the US.

## Methods

The study used data from TriNetX, a global federated health research network that provided an anonymized dataset of electronic medical records (EMRs). The TriNetX network was searched on June 9, 2020, and a deidentified dataset of patients with COVID-19 aged up to 90 years identified in EMRs between January 20, 2020, and May 26, 2020, was provided. The data on the research network come from academic medical centers, specialty physician practices, and community hospitals. Further details about TriNetX processes and standardization of data are in S1 Text.

Patients with COVID-19 were identified following criteria provided by TriNetX based on Centers for Disease Control and Prevention (CDC) coding guidelines [15]. Patients were included if they had 1 or more of the following International Classification of Diseases, Ninth Revision and Tenth Revision, Clinical Modification (ICD-10-CM) codes in their EMRs: U07.1 COVID-19; B97.29 Other coronavirus as the cause of diseases classified elsewhere; B34.2 Coronavirus infection, unspecified; or a positive test result identified with COVID-19-specific laboratory Logical Observation Identifiers Names and Codes (LOINCs). The code U07.2 COVID-19, virus not identified, was also searched for, but no patients were found to have this code recorded. Patients with ICD-9 code 079.89 were excluded to reduce the likelihood of patients with false positive COVID-19 because this code may still be used occasionally as a "catch-all” code for >50 viral infections.

The study included all patients with COVID-19 recorded in their EMRs from participating healthcare organizations. This included both inpatient and outpatient care settings, but the type of visit was not well recorded. History of comorbidities listed in the Charlson comorbidity index were identified if the patient had a corresponding ICD code for the condition since January 1, 2015, in their EMRs captured in TriNetX [16]. The timeframe was chosen based on a previous study that examined comorbidities in a 5-year interval [17]. Deaths during an inpatient or outpatient visit at the participating healthcare organizations were recorded; however, deaths occurring outside of the hospital setting are not well captured. As date of death was not available in the downloaded deidentified dataset from TriNetX, we estimated date of death based on the most recent date recorded in the patient EMRs from the following: diagnosis (date), procedure (date), encounter (end date), vital signs (date), medication (start date). We estimated time to mortality following COVID-19 using the estimated date of death minus the first recording of COVID-19 in the EMRs (either from a positive laboratory test result or ICD-10-CM code).

Descriptive statistics included proportions for categorical variables and medians and interquartile ranges (IQRs) for continuous variables. Unadjusted and multivariate logistic regressions were performed to explore associations between age, sex, ethnicity, comorbidities, and mortality. Any deaths during the study period captured in EMRs of the participating healthcare organizations were included in the analysis. Variables identified as statistically significant predictors with a significance level of *p* < 0.05 were planned to be inserted into a forward multivariate logistic model. All variables were statistically significant in unadjusted analysis and inserted to the multivariate model, apart from in age-stratified analyses. No imputations were made for missing data. Data were requested from TriNetX and all analyses were conducted with Stata v.14.0.

This study is reported as per the Strengthening the Reporting of Observational Studies in Epidemiology (STROBE) guidelines (S1 STROBE Checklist) [18]. No published prospective analysis plan was produced. Analyses were planned prior to download of the study data from TriNetX. Upon receipt of the data, we were informed that date of death was not available, because of data privacy agreements, and therefore planned Cox proportional hazard models were changed to logistic regression models. We also changed the search to only include people with COVID-19 coded in their EMRs 2 weeks before the search date, to allow a 2-week time window for potential follow-up and to be comparable with a cohort study which examined comorbidities and mortality with COVID-19 in the United Kingdom [19]. We further limited analyses to people aged 18 years or over and performed age-stratified analyses. We did not impose any further exclusion criteria to limit selection bias.

### Ethics statement

No ethical approval was obtained, as no patient identifiable information was received and the data were analyzed anonymously.

## Results

In total 33,488 patients from 24 healthcare organizations had 1 or more of the specified COVID-19 codes or a positive laboratory test in their EMRs during the study period. Patients in the cohort were distributed between the 4 large Census Bureau–designated regions of the US as follows: 27% (*n* = 9,040) in the Northeast, 21% (*n* = 6,898) in the Midwest, 30% (*n* = 10,192) in the South, 21% (*n* = 7,181) in the West, and 0.5% (*n* = 177) unknown. Of the patients identified, 50.3% (*n* = 16,833) were identified from ICD codes only (U07.1, B97.29, or B34.2); 15.2% (*n* = 5,096) were identified from the ICD codes B97.29 or B34.2 only.

Of the total number of patients, 2.0% (*n* = 670) did not have age or sex recorded within the TriNetX network and were excluded from analyses. A further 1,357 patients were aged <18 years and were also excluded. Therefore, 31,461 patients were included in analyses. The median (IQR) age was 50 (35–63) years and 54.5% (*n* = 17,155) were female. In total, 45.2% (*n* = 14,225) of participants were white, according to their EMRs, and 27.8% (*n* = 8,758) were black or African American; smaller proportions of people from other ethnicities were identified, and ethnicity was unknown for 23.8% (*n* = 7,476). Of the total cohort, 59.5% (18,734) of patients did not have a record of any of the comorbidities listed in the Charlson comorbidity index; 17.4% (*n* = 5,458) had 1 comorbidity listed, 7.9% (*n* = 2,473) had 2 comorbidities, and 15.2% (*n* = 4,796) had ≥3 comorbidities. The most common comorbidities were chronic pulmonary disease (17.5%, *n* = 5,513) and diabetes mellitus (15.0%, *n* = 4,710) (Table 1).

**Table 1:** Baseline characteristics of patients with COVID-19 in the TriNetX research network as of May 26, 2020.

Characteristics	All participants (*n* = 31,461)	Deceased (*n* = 1,296)	Alive (*n* = 30,165)
Age, median (IQR), years	50 (35–63)	72 (63–80)	49 (35–62)
<50	49.5 (15,578)	8.0 (104)	51.3 (15,474)
50–59	18.6 (5,855)	11.2 (145)	18.9 (5,710)
60–69	15.4 (4,843)	22.0 (285)	15.1 (4,558)
70–79	9.6 (3,015)	30.8 (399)	8.7 (2,616)
80–90	6.9 (2,170)	28.0 (363)	6.0 (1,807)
Sex			
Female	54.5 (17,155)	41.1 (532)	55.1 (16,623)
Male	45.5 (14,306)	59.0 (764)	44.9 (13,542)
Ethnicity			
White	45.2 (14,225)	48.5 (629)	45.1 (13,596)
Black or African American	27.8 (8,758)	39.0 (506)	27.4 (8,252)
Asian	2.5 (791)	1.2 (16)	2.6 (775)
Native Hawaiian or other Pacific Islander	0.4 (115)	0.7 (9)	0.4 (106)
American Indian or Alaska Native	0.3 (96)	0	0.3 (96)
Unknown	23.8 (7,476)	10.5 (136)	24.3 (7,340)
Weighted Charlson comorbidity index			
0	59.5 (18,734)	17.3 (224)	61.4 (18,510)
1	17.4 (5,458)	16.1 (208)	17.4 (5,250)
2	7.9 (2,473)	14.4 (187)	7.6 (2,286)
≥3	15.2 (4,796)	52.2 (677)	13.7 (4,119)
Comorbidities within the Charlson comorbidity index			
Myocardial infarction	4.1 (1,280)	20.2 (262)	3.4 (1,018)
Congestive heart failure	7.3 (2,297)	30.8 (399)	6.3 (1,898)
Peripheral vascular disease	5.1 (1,601)	18.2 (236)	4.5 (1,365)
Cerebrovascular disease	6.1 (1,922)	19.6 (254)	5.5 (1,668)
Dementia	3.3 (1,031)	15.4 (199)	2.8 (832)
Chronic pulmonary disease	17.5 (5,513)	30.2 (391)	17.0 (5,122)
Rheumatic disease	2.2 (681)	4.2 (54)	2.1 (627)
Peptic ulcer disease	1.4 (432)	2.9 (37)	1.3 (395)
Mild liver disease	4.8 (1,497)	9.3 (121)	4.6 (1,376)
Moderate/severe liver disease	0.4 (138)	1.7 (22)	0.4 (116)
Diabetes mellitus	15.0 (4,710)	32.4 (420)	14.2 (4,290)
Hemiplegia or paraplegia	1.3 (421)	3.0 (39)	1.3 (382)
Renal disease	8.7 (2,735)	37.5 (486)	7.5 (2,249)
Any malignancy^a^	6.3 (1,966)	14.8 (192)	5.9 (1,774)
Metastatic solid tumor	1.2 (383)	3.9 (51)	1.1 (332)
AIDS/HIV	0.7 (226)	1.3 (17)	0.7 (209)

Results are % (*n*) unless otherwise stated.

^a^Any malignancy, including lymphoma and leukemia, except malignant neoplasm of skin. Comorbidities are based on ICD-10 codes from 2015.

Abbreviations: COVID-19, coronavirus disease 2019; IQR, interquartile range.

Of the 31,461 patients, 5.3% (*n* = 1,669) had no end date recorded on a healthcare organization encounter during the study period and therefore may have not yet been discharged. The median (IQR) estimated time in the study was 54 days (36–68). During the study period, 4.1% (*n* = 1,296) patients were recorded as deceased, and the estimated median (IQR) time to mortality after first recording of COVID-19 was 9 days (4–17). Of the total patient cohort, 4.1% (*n* = 1,296) had a procedure code indicating invasive mechanical ventilation during the study period in EMRs. Of the patients who died, 49.5% (*n* = 642) had a procedure code for invasive mechanical ventilation compared to 2.2% (*n* = 654) of patients who were not recorded as deceased.

In unadjusted analyses, the following variables were associated with higher odds of death: older age, male sex, being black or African American compared to white, and all comorbidities investigated (Table 2). Being Asian or having unknown ethnicity compared to being white was associated with lower odds of death. On multivariate analyses, higher odds of death were associated with older age (odds ratio [OR] per year 1.063; 95% confidence interval [CI] 1.058–1.068; *p* < 0.001), male sex (OR 1.75; 95% CI 1.55–1.98; *p* < 0.001), being black or African American compared to white (OR 1.50; 95% CI 1.31–1.71; *p* < 0.001), myocardial infarction (OR 1.97; 95% CI 1.64–2.35; *p* < 0.001), congestive heart failure (OR 1.42; 95% CI 1.21–1.67; *p* < 0.001), dementia (OR 1.29; 95% CI 1.07–1.56; *p* = 0.008), chronic pulmonary disease (OR 1.24; 95% CI 1.08–1.43; *p* = 0.003), mild liver disease (OR 1.26; 95% CI 1.00–1.59; *p* = 0.046), moderate/severe liver disease (OR 2.62; 95% CI 1.53–4.47; *p* < 0.001), renal disease (OR 2.13; 95% CI 1.84–2.46; *p* < 0.001), and metastatic solid tumor (OR 1.70; 95% CI 1.19–2.43; *p* = 0.004). Being native Hawaiian or other Pacific Islander was associated with higher odds of death compared to being white, but there were only 115 people identified as native Hawaiian or other Pacific Islander in this cohort. Having an unknown ethnicity was associated with reduced odds of death compared to being white (OR 0.74; 95% CI 0.60–0.90, *p* = 0.003).

**Table 2:** Unadjusted and multivariate analysis of factors associated with mortality in adults with COVID-19 coded in the TriNetX research network as of May 26, 2020 (*n* = 31,461).

	Unadjusted results		Multivariate results	
Characteristics	Death with COVID-19, OR (95% CI)	*p*-Value	Death with COVID-19, OR (95% CI)	*p*-Value
Age (per year)	1.074 (1.069–1.078)	<0.001	1.063 (1.058–1.068)	<0.001
Male sex	1.76 (1.57–1.97)	<0.001	1.75 (1.55–1.98)	<0.001
Ethnicity				
White	Ref		Ref	
Black or African American	1.33 (1.18–1.49)	<0.001	1.50 (1.31–1.71)	<0.001
Asian	0.45 (0.27–0.74)	0.002	0.60 (0.36–1.01)	0.06
Native Hawaiian or other Pacific Islander	1.84 (0.92–3.64)	0.09	3.63 (1.75–7.52)	0.001
American Indian or Alaska Native	-	-	-	-
Unknown	0.40 (0.33–0.48)	<0.001	0.74 (0.60–0.90)	0.003
Comorbidities within the Charlson comorbidity index				
Myocardial infarction	7.25 (6.25–8.42)	<0.001	1.97 (1.64–2.35)	<0.001
Congestive heart failure	6.62 (5.84–7.52)	<0.001	1.42 (1.21–1.67)	<0.001
Peripheral vascular disease	4.70 (4.04–5.46)	<0.001	0.89 (0.74–1.07)	0.20
Cerebrovascular disease	4.16 (3.60–4.82)	<0.001	1.07 (0.90–1.28)	0.44
Dementia	6.40 (5.42–7.55)	<0.001	1.29 (1.07–1.56)	0.008
Chronic pulmonary disease	2.11 (1.87–2.39)	<0.001	1.24 (1.08–1.43)	0.003
Rheumatic disease	2.05 (1.54–2.72)	<0.001	1.17 (0.85–1.60)	0.16
Peptic ulcer disease	2.21 (1.57–3.12)	<0.001	0.76 (0.52–1.12)	0.21
Mild liver disease	2.15 (1.77–2.62)	<0.001	1.26 (1.00–1.59)	0.046
Moderate/severe liver disease	4.47 (2.83–7.08)	<0.001	2.62 (1.53–4.47)	<0.001
Diabetes mellitus	2.89 (2.56–3.26)	<0.001	1.11 (0.96–1.27)	0.16
Hemiplegia or paraplegia	2.42 (1.73–3.38)	<0.001	0.76 (0.52–1.09)	0.14
Renal disease	7.45 (6.60–8.40)	<0.001	2.13 (1.84–2.46)	<0.001
Any malignancy	2.78 (2.37–3.27)	<0.001	0.87 (0.72–1.06)	0.17
Metastatic solid tumor	3.68 (2.73–4.97)	<0.001	1.70 (1.19–2.43)	0.004
AIDS/HIV	1.91 (1.16–3.13)	0.011	1.71 (1.00–2.93)	0.051

American Indian or Alaska Native omitted because there were no deaths among this group. Only characteristics *p* < 0.05 in the unadjusted analyses were included in the multivariate analysis.

Abbreviations: CI, confidence interval; COVID-19, coronavirus disease 2019; OR, odds ratio.

### Age-stratified analyses

Differences between age groups were found when results were stratified by age group (<50 years, 50–69 years, or 70–90 years). For all age groups, older age, being male, and being black or African American compared to being white were associated with higher odds of death in multivariate analyses. When examining comorbidities, history of myocardial infarction and renal disease were associated with higher odds of death for all age groups, but there were differences across age groups for other comorbidities and associations with mortality. For people aged <50 years, history of mild liver disease and any malignancy were associated with higher odds of death. For people aged 50–69 years, history of congestive heart failure, chronic pulmonary disease, moderate/severe liver disease, metastatic solid tumor, and AIDS/HIV were all associated with higher odds of death. For people aged 70–90 years, history of congestive heart failure and dementia were associated with higher odds of death (S1 Table, S2 Table and S3 Table).

## Discussion

In this retrospective cohort study, we identified 31,461 adults with confirmed COVID-19 from EMRs from 24 healthcare organizations in the US between January 20, 2020, and May 26, 2020. Older age, male sex, black or African American ethnicity, and a history of myocardial infarction, congestive heart failure, dementia, chronic pulmonary disease, mild liver disease, moderate/severe liver disease, renal disease, and metastatic solid tumor were associated with higher odds of mortality after adjustment for these factors and other comorbidities.

Older age has been frequently reported to be an important factor associated with disease severity or mortality in patients with COVID-19 [4–6]. There is some previous evidence to suggest men, people who are black or African American, or people from certain ethnic minority backgrounds may also be higher risk for COVID-19 [7–10]. Underlying comorbidities including preexisting concurrent cardiovascular or cerebrovascular diseases, hypertension, and diabetes mellitus have been reported as highly prevalent in studies of COVID-19 patients and/or associated with poorer outcomes for these patients [5, 7, 11]. This study further suggests that cardiovascular conditions such as previous myocardial infarction or congestive heart failure may be important factors influencing mortality in patients with COVID-19. Furthermore, a recent meta-analysis of 4 studies suggested chronic kidney disease may be associated with enhanced risk of severe COVID-19 infection [12]. The results of this study agree with a recent UK-based cohort study that showed an association between older age, male sex, cardiac disease, non-asthmatic pulmonary disease, kidney disease, liver disease, malignancy, and dementia and higher mortality in hospital for patients with COVID-19 [19]. The UK-based study also showed an association between obesity and higher mortality, which could not be explored in the current study because of a paucity of data on body mass index.

Age-stratified analyses in the current study highlighted the potential importance of considering age groups when examining associations between comorbidities and mortality with COVID-19. We found a history of myocardial infarction or renal disease to be associated with mortality in all age groups, but there were differences found between age groups for which other comorbidities associated with mortality. In this study, a borderline association was found in this study between AIDS/HIV and mortality with COVID-19 when examining all adults, but age-stratified analyses suggested AIDS/HIV was associated with significantly higher odds of mortality only in people aged 50–69 years. Patients with AIDS/HIV represented a small proportion of the total patient population, and larger studies of patients with AIDS/HIV and COVID-19 are needed to further determine the association. The findings in this study of an association between liver disease and mortality for patients with COVID-19 are in line with a recently published study that used data from TriNetX and showed patients with preexisting liver disease had a higher risk of mortality with COVID-19 [20].

### Limitations

This study has several limitations. The data were collected from healthcare organization EMR databases and some comorbidities may be underreported, and ethnicity was not available for all participants. Residual confounding may include lifestyle factors and socioeconomic status, which were not available from EMRs. Body mass index was only available for approximately 5% of participants and so was not included in analyses. We could also not determine the influence of attending different healthcare organizations, owing to data privacy restrictions. In these analyses, we found a significant association between those with “unknown” ethnicity from EMRs and reduced odds of death compared to white patients. Participants with unknown ethnicity may have been participants who did not fit in the limited prespecified ethnicity categories within TriNetX, but this could not be explored further, given restrictions on data privacy. Only age at death, not date of death, was available in downloaded data, so time-to-event analyses could not be performed. Participants who died with COVID-19 after the study end date would have been recorded as alive in the present analyses, and deaths outside of the participating healthcare organizations are not well captured. The data in EMRs are susceptible to errors in coding or data entry when patient information is translated to ICD codes. In this study, we looked at any history of the comorbidities identified in the Charlson comorbidity index since 2015. We could not determine if the patient was no longer living with the condition. Recording of ICD codes in administrative data may vary by factors such as age, number of comorbidities, severity of illness, length of hospitalization, and whether in-hospital death occurred [21]. The data were from multiple healthcare organizations in the US but may not be representative of the wider US population, and the generalizability of the results beyond this cohort is unclear.

## Conclusions

Increasing age, being male, being black or African American compared to white, and a history of myocardial infarction, congestive heart failure, dementia, chronic pulmonary disease, liver disease, renal disease, and metastatic solid tumor were associated with mortality in adults with COVID-19. There were differences in which comorbidities were associated with higher odds of mortality depending on age group.

## Supporting information

S1 STROBE Checklist STROBE checklist. STROBE, Strengthening the Reporting of Observational Studies in Epidemiology.
(DOCX)

S1 Table Unadjusted and multivariate analysis of factors associated with mortality in adults aged <50 years with COVID-19 coded in the TriNetX research network as of May 26, 2020 (*n* = 15,578). COVID-19, coronavirus disease 2019.
(DOCX)

S2 Table Unadjusted and multivariate analysis of factors associated with mortality in adults aged 50–69 years with COVID-19 coded in the TriNetX research network as of May 26, 2020 (*n* = 10,698). COVID-19, coronavirus disease 2019.
(DOCX)

S3 Table Unadjusted and multivariate analysis of factors associated with mortality in adults aged 70–90 years with COVID-19 coded in the TriNetX research network as of May 26, 2020 (*n* = 5,185). COVID-19, coronavirus disease 2019.
(DOCX)

S1 Text TriNetX standardization of data. (DOCX)

S1 Data (XLS)

## Abbreviations

CDC: Centers for Disease Control and Prevention
CI: confidence interval
COVID-19: coronavirus disease 2019
EMR: electronic medical record
ICD: International Classification of Diseases
IQR: interquartile range
LOINC: Logical Observation Identifiers Names and Codes
OR: odds ratio
STROBE: Strengthening the Reporting of Observational Studies in Epidemiology
