# Supplementary material for: Comorbidities associated with mortality in 31,461 adults with COVID-19 in the United States: A federated electronic medical record analysis
Source: PLoS Med. 2020 Sep 10;17(9):e1003321. doi: 10.1371/journal.pmed.1003321 (PMC7482833; doi:10.1371/journal.pmed.1003321)
Supplement: S1 STROBE Checklist — STROBE, Strengthening the Reporting of Observational Studies in Epidemiology. (DOCX) [file pmed.1003321.s001.docx]

STROBE Statement—checklist of items that should be included in reports of observational studies

|  | Item No. | Recommendation | Page  No. | Relevant text from manuscript |
| --- | --- | --- | --- | --- |
| **Title and abstract** | 1 | (*a*) Indicate the study’s design with a commonly used term in the title or the abstract | Abstract, second paragraph | “Retrospective cohort study” |
|  |  | (*b*) Provide in the abstract an informative and balanced summary of what was done and what was found | Abstract, second paragraph |  |
| Introduction | | | |  |
| Background/rationale | 2 | Explain the scientific background and rationale for the investigation being reported | Introduction, paragraphs 1-3 |  |
| Objectives | 3 | State specific objectives, including any prespecified hypotheses | Introduction, paragraph 3 | “The objective of the study was to determine associations between age, sex, race, co-morbidities and mortality of adults with COVID-19 in the US.” |
| Methods | | | |  |
| Study design | 4 | Present key elements of study design early in the paper | Methods, paragraph 1 |  |
| Setting | 5 | Describe the setting, locations, and relevant dates, including periods of recruitment, exposure, follow-up, and data collection | Methods, paragraph 1 | “The TriNetX network was searched on June 9, 2020 and a de-identified dataset of patients with COVID-19 aged up to 90 years old identified in EMRs between January 20, 2020 and May 26, 2020 was provided. The data on the research network comes from academic medical centers, specialty physician practices and hospitals. Further details about TriNetX processes and standardization of data are in the supplementary methods.” |
| Participants | 6 | (*a*) *Cohort study*—Give the eligibility criteria, and the sources and methods of selection of participants. Describe methods of follow-up  *Case-control study*—Give the eligibility criteria, and the sources and methods of case ascertainment and control selection. Give the rationale for the choice of cases and controls  *Cross-sectional study*—Give the eligibility criteria, and the sources and methods of selection of participants | Methods, paragraphs 2 and 5 | “Patients with COVID-19 were identified following criteria provided by TriNetX based on Centers for Disease Control (CDC) coding guidelines (15). Patients were included if they had one or more of the following International Classification of Diseases, Ninth Revision and Tenth Revision, Clinical Modification (ICD-10-CM) codes in their EMRs: U07.1 COVID-19; B97.29 Other coronavirus as the cause of diseases classified elsewhere; B34.2 Coronavirus infection, unspecified; or a positive test result identified with COVID-19 specific laboratory Logical Observation Identifiers Names and Codes (LOINCs). The code U07.2 COVID-19, virus not identified, was also searched for, but no patients were found to have this code recorded. Patients with ICD-9 code 079.89 (mapped to ICD-10 code B34.2 and B97.2) were excluded to reduce the likelihood of patients with false positive COVID-19 because this code may still be used occasionally as a "catch-all' code for >50 viral infections.” And “We also changed the search to only include people with COVID-19 coded in their EMRs two weeks before the search date, to allow a two-week time window for potential follow-up and to be comparable with a cohort study which examined co-morbidities and mortality with COVID-19 in the UK (19). We further limited analyses to people aged 18 years or over..” |
|  |  | (*b*) *Cohort study*—For matched studies, give matching criteria and number of exposed and unexposed  *Case-control study*—For matched studies, give matching criteria and the number of controls per case | N/a |  |
| Variables | 7 | Clearly define all outcomes, exposures, predictors, potential confounders, and effect modifiers. Give diagnostic criteria, if applicable | Methods, paragraph 4 | “Unadjusted and multivariate logistic regressions were performed to explore associations between age, sex, race, co-morbidities and mortality.” |
| Data sources/ measurement | 8* | For each variable of interest, give sources of data and details of methods of assessment (measurement). Describe comparability of assessment methods if there is more than one group | Methods, paragraph 3 | “History of co-morbidities listed in the Charlson co-morbidity index were identified if the patient had a corresponding ICD code for the condition since January 1, 2015 in their EMRs captured in TriNetX (16).” |
| Bias | 9 | Describe any efforts to address potential sources of bias | Methods, paragraph 5 | “We did not impose any further exclusion criteria to limit selection bias.” |
| Study size | 10 | Explain how the study size was arrived at | Methods, paragraph 1 and results paragraph 1 and 2 | “The TriNetX network was searched on June 9, 2020 and a de-identified dataset of patients with COVID-19 aged up to 90 years old identified in EMRs between January 20, 2020 and May 26, 2020 was provided.”  “In total 33,488 patients from 24 healthcare organizations had one or more of the specified COVID-19 codes or a positive laboratory test in their EMRs during the study period.”  “Of the total number of patients, 2.0% (n=670) did not have age or sex recorded within the TriNetX network and were excluded from analyses. A further 1,357 patients were aged <18 years old and were also excluded. Therefore, 31,461 patients were included in analyses.” |

Continued on next page

| Quantitative variables | 11 | Explain how quantitative variables were handled in the analyses. If applicable, describe which groupings were chosen and why | Methods, paragraph 3 |  |
| --- | --- | --- | --- | --- |
| Statistical methods | 12 | (*a*) Describe all statistical methods, including those used to control for confounding | Methods, paragraph 4 |  |
|  |  | (*b*) Describe any methods used to examine subgroups and interactions | Methods, paragraph 5 | “…and performed age-stratified analyses.” |
|  |  | (*c*) Explain how missing data were addressed | Methods, paragraph 4 | “No imputations were made for missing data.” |
|  |  | (*d*) *Cohort study*—If applicable, explain how loss to follow-up was addressed  *Case-control study*—If applicable, explain how matching of cases and controls was addressed  *Cross-sectional study*—If applicable, describe analytical methods taking account of sampling strategy | N/a |  |
|  |  | (*e*) Describe any sensitivity analyses | N/a |  |
| Results | | | | |
| Participants | 13* | (a) Report numbers of individuals at each stage of study—eg numbers potentially eligible, examined for eligibility, confirmed eligible, included in the study, completing follow-up, and analysed | Results, paragraphs 1 and 2 | “In total 33,488 patients from 24 healthcare organizations had one or more of the specified COVID-19 codes or a positive laboratory test in their EMRs during the study period.”  “Of the total number of patients, 2.0% (n=670) did not have age or sex recorded within the TriNetX network and were excluded from analyses. A further 1,357 patients were aged <18 years old and were also excluded. Therefore, 31,461 patients were included in analyses.” |
|  |  | (b) Give reasons for non-participation at each stage | N/a |  |
|  |  | (c) Consider use of a flow diagram | N/a |  |
| Descriptive data | 14* | (a) Give characteristics of study participants (eg demographic, clinical, social) and information on exposures and potential confounders | Results, paragraph 2 and Table 1 |  |
|  |  | (b) Indicate number of participants with missing data for each variable of interest | Results, paragraph 2 | “..race was unknown for 23.8% (n=7,476).” |
|  |  | (c) *Cohort study*—Summarise follow-up time (eg, average and total amount) | Results, paragraph 1 | “The median (IQR) estimated time in the study was 54 days (36-68).” |
| Outcome data | 15* | *Cohort study*—Report numbers of outcome events or summary measures over time | Results, paragraph 2 | “During the study period, 4.1% (n=1,296) patients were recorded as deceased and the estimated median (interquartile range) time to mortality after first recording of COVID-19 was 9 days (4-17).” |
|  |  | *Case-control study—*Report numbers in each exposure category, or summary measures of exposure |  |  |
|  |  | *Cross-sectional study—*Report numbers of outcome events or summary measures |  |  |
| Main results | 16 | (*a*) Give unadjusted estimates and, if applicable, confounder-adjusted estimates and their precision (eg, 95% confidence interval). Make clear which confounders were adjusted for and why they were included | Table 2 |  |
|  |  | (*b*) Report category boundaries when continuous variables were categorized | N/a |  |
|  |  | (*c*) If relevant, consider translating estimates of relative risk into absolute risk for a meaningful time period | N/a |  |

Continued on next page

| Other analyses | 17 | Report other analyses done—eg analyses of subgroups and interactions, and sensitivity analyses | Supplementary tables 1-3 |  |
| --- | --- | --- | --- | --- |
| Discussion | | | | |
| Key results | 18 | Summarise key results with reference to study objectives | Discussion, paragraph 1 |  |
| Limitations | 19 | Discuss limitations of the study, taking into account sources of potential bias or imprecision. Discuss both direction and magnitude of any potential bias | Discussion, paragraph 4 |  |
| Interpretation | 20 | Give a cautious overall interpretation of results considering objectives, limitations, multiplicity of analyses, results from similar studies, and other relevant evidence | Discussion paragraphs 2-3 |  |
| Generalisability | 21 | Discuss the generalisability (external validity) of the study results | Discussion, paragraph 4 | “The data were from multiple healthcare organizations in the US but may not be representative of the wider US population and the generalisability of the results beyond this cohort is unclear.” |
| Other information | |  | | |
| Funding | 22 | Give the source of funding and the role of the funders for the present study and, if applicable, for the original study on which the present article is based | Online submission form. |  |

*Give information separately for cases and controls in case-control studies and, if applicable, for exposed and unexposed groups in cohort and cross-sectional studies.

**Note:** An Explanation and Elaboration article discusses each checklist item and gives methodological background and published examples of transparent reporting. The STROBE checklist is best used in conjunction with this article (freely available on the Web sites of PLoS Medicine at http://www.plosmedicine.org/, Annals of Internal Medicine at http://www.annals.org/, and Epidemiology at http://www.epidem.com/). Information on the STROBE Initiative is available at www.strobe-statement.org.
