# Supplementary material for: Comorbidities associated with mortality in 31,461 adults with COVID-19 in the United States: A federated electronic medical record analysis
Source: PLoS Med. 2020 Sep 10;17(9):e1003321. doi: 10.1371/journal.pmed.1003321 (PMC7482833; doi:10.1371/journal.pmed.1003321)
Supplement: S1 Table — COVID-19, coronavirus disease 2019. (DOCX) [file pmed.1003321.s002.docx]

S1 Table. Unadjusted and multivariate analysis of factors associated with mortality in adults aged <50 with COVID-19 coded in the TriNetX research network as of May 26, 2020 (n=15,578).

|  | **Unadjusted results** | | **Multivariate results** | |
| --- | --- | --- | --- | --- |
| Characteristics | Death with COVID-19, OR (95%CI) | P-value | Death with COVID-19, OR (95%CI) | P-value |
| Age (per year) | 1.06 (1.04, 1.09) | <0.001 | 1.03 (1.01, 1.06) | 0.015 |
| Male Sex | 1.89 (1.28, 2.79) | <0.001 | 1.91 (1.27, 2.89) | 0.002 |
| Ethnicity |  |  |  |  |
| White | Ref |  | Ref |  |
| Black or African American | 3.88 (2.42, 6.20) | <0.001 | 3.37 (2.06, 5.54) | <0.001 |
| Asian | 0.62 (0.08, 4.57) | 0.638 | 0.71 (0.10, 5.31) | 0.74 |
| Native Hawaiian or other Pacific Islander | 4.78 (0.64, 36.0) | 0.13 | 4.84 (0.61, 38.7) | 0.14 |
| American Indian or Alaska Native | - | - | - | - |
| Unknown | 1.06 (0.59, 1.93) | 0.840 | 1.17 (0.63, 2.16) | 0.62 |
| Co-morbidities within the Charlson co-morbidity index | |  |  |  |
| Myocardial Infarction | 15.9 (8.3, 30.6) | <0.001 | 2.52 (1.09, 5.84) | 0.032 |
| Congestive Heart Failure | 11.7 (6.84, 19.9) | <0.001 | 1.61 (0.77, 3.39) | 0.208 |
| Peripheral Vascular Disease | 17.0 (8.6, 33.7) | <0.001 | 2.35 (0.97, 5.73) | 0.06 |
| Cerebrovascular Disease | 5.20 (2.38, 11.3) | <0.001 | 0.79 (0.29, 2.14) | 0.64 |
| Dementia | - | - | - | - |
| Chronic Pulmonary Disease | 2.01 (1.28, 3.15) | <0.001 | 1.43 (0.86, 2.35) | 0.17 |
| Rheumatic Disease | 2.30 (0.72, 7.32) | 0.16 | - | - |
| Peptic Ulcer Disease | 4.48 (1.40, 14.3) | 0.012 | 0.78 (0.18, 3.42) | 0.74 |
| Mild Liver Disease | 6.00 (3.49, 10.3) | <0.001 | 2.35 (1.19, 4.65) | 0.014 |
| Moderate/Severe Liver Disease | 13.1 (3.97, 43.3) | <0.001 | 2.18 (0.51, 9.25) | 0.29 |
| Diabetes without Chronic Complications | 3.69 (2.28, 5.99) | <0.001 | 0.95 (0.52, 1.73) | 0.87 |
| Hemiplegia or Paraplegia | 9.37 (3.72, 23.6) | <0.001 | 3.32 (0.97, 11.31) | 0.06 |
| Renal Disease | 16.0 (10.1, 25.3) | <0.001 | 4.38 (2.33, 8.25) | <0.001 |
| Any Malignancy | 4.02 (1.85, 8.73) | <0.001 | 2.49 (1.01, 6.19) | 0.049 |
| Metastatic Solid Tumour | - | - | - | - |
| AIDS/HIV | 4.39 (1.37, 14.1) | 0.013 | 1.32 (0.36, 4.88) | 0.68 |

CI: confidence interval, OR: Odds Ratio. American Indian or Alaska Native omitted because there were no deaths among this group. N=9 adults aged <50 had dementia recorded in their electronic medical records and none died; N=54 adults aged<50 had metastatic solid tumour recorded in their electronic medical records and none died. Only characteristics p<0.05 in the unadjusted analyses were included in the multivariate analysis.
