# Supplementary material for: Comorbidities associated with mortality in 31,461 adults with COVID-19 in the United States: A federated electronic medical record analysis
Source: PLoS Med. 2020 Sep 10;17(9):e1003321. doi: 10.1371/journal.pmed.1003321 (PMC7482833; doi:10.1371/journal.pmed.1003321)
Supplement: S2 Table — COVID-19, coronavirus disease 2019. (DOCX) [file pmed.1003321.s003.docx]

S2 Table. Unadjusted and multivariate analysis of factors associated with mortality in adults aged 50-69 with COVID-19 coded in the TriNetX research network as of May 26, 2020 (n=10,698).

|  | **Unadjusted results** | | **Multivariate results** | |
| --- | --- | --- | --- | --- |
| Characteristics | Death with COVID-19, OR (95%CI) | P-value | Death with COVID-19, OR (95%CI) | P-value |
| Age (per year) | 1.10 (1.08, 1.12) | <0.001 | 1.07 (1.07, 1.10) | <0.001 |
| Male Sex | 1.89 (1.55, 2.31) | <0.001 | 1.81 (1.47, 2.24) | <0.001 |
| Ethnicity |  |  |  |  |
| White | Ref |  | Ref |  |
| Black or African American | 1.48 (1.18, 1.79) | <0.001 | 1.37 (1.10, 1.72) | 0.005 |
| Asian | 0.31 (0.10, 0.98) | 0.047 | 0.40 (0.13, 1.29) | 0.13 |
| Native Hawaiian or other Pacific Islander | 3.28 (1.38, 7.77) | 0.007 | 4.56 (1.88, 11.0) | 0.001 |
| American Indian or Alaska Native | - | - | - | - |
| Unknown | 0.60 (0.43, 0.82) | 0.002 | 0.75 (0.54, 1.04) | 0.09 |
| Co-morbidities within the Charlson co-morbidity index | |  |  |  |
| Myocardial Infarction | 4.67 (3.58, 6.09) | <0.001 | 1.86 (1.35, 2.57) | <0.001 |
| Congestive Heart Failure | 4.62 (3.68, 5.78) | <0.001 | 1.62 (1.21, 2.18) | 0.001 |
| Peripheral Vascular Disease | 3.11 (2.36, 4.10) | <0.001 | 0.98 (0.71, 1.36) | 0.90 |
| Cerebrovascular Disease | 2.44 (1.85, 3.22) | <0.001 | 1.09 (0.78, 1.51) | 0.61 |
| Dementia | 2.65 (1.49, 4.75) | 0.001 | 1.21 (0.64, 2.26) | 0.55 |
| Chronic Pulmonary Disease | 1.87 (1.52, 2.31) | <0.001 | 1.35 (1.07, 1.71) | 0.012 |
| Rheumatic Disease | 1.77 (1.12, 2.78) | 0.014 | 1.18 (0.72, 1.93) | 0.51 |
| Peptic Ulcer Disease | 1.50 (0.81, 2.77) | 0.200 | - | - |
| Mild Liver Disease | 2.50 (1.91, 3.27) | <0.001 | 1.27 (0.92, 1.75) | 0.15 |
| Moderate/Severe Liver Disease | 6.75 (3.97, 11.48) | <0.001 | 3.05 (1.61, 5.76) | 0.001 |
| Diabetes without Chronic Complications | 2.07 (1.70, 2.54) | <0.001 | 1.17 (0.93, 1.47) | 0.18 |
| Hemiplegia or Paraplegia | 2.44 (1.45, 4.12) | 0.001 | 0.89 (0.49, 1.61) | 0.70 |
| Renal Disease | 4.88 (3.95, 6.02) | <0.001 | 2.23 (1.72, 2.89) | <0.001 |
| Any Malignancy | 1.74 (1.31, 2.31) | <0.001 | 0.83 (0.58, 1.20) | 0.32 |
| Metastatic Solid Tumour | 3.20 (2.01, 5.10) | <0.001 | 2.28 (1.27, 4.11) | 0.006 |
| AIDS/HIV | 3.07 (1.63, 5.80) | 0.001 | 2.36 (1.21, 4.60) | 0.012 |

CI: confidence interval, OR: Odds Ratio. American Indian or Alaska Native omitted because there were no deaths among this group. Only characteristics p<0.05 in the unadjusted analyses were included in the multivariate analysis.
