# Supplementary material for: Comorbidities associated with mortality in 31,461 adults with COVID-19 in the United States: A federated electronic medical record analysis
Source: PLoS Med. 2020 Sep 10;17(9):e1003321. doi: 10.1371/journal.pmed.1003321 (PMC7482833; doi:10.1371/journal.pmed.1003321)
Supplement: S3 Table — COVID-19, coronavirus disease 2019. (DOCX) [file pmed.1003321.s004.docx]

Supplementary Table 3. Unadjusted and multivariate analysis of factors associated with mortality in adults aged 70-90 years with COVID-19 coded in the TriNetX research network as of May 26, 2020 (n=5,185).

|  | **Unadjusted results** | | **Multivariate results** | |
| --- | --- | --- | --- | --- |
| Characteristics | Death with COVID-19, OR (95%CI) | P-value | Death with COVID-19, OR (95%CI) | P-value |
| Age (per year) | 1.03 (1.01, 1.04) | <0.001 | 1.02 (1.01, 1.04) | 0.002 |
| Male Sex | 1.58 (1.35, 1.85) | <0.001 | 1.60 (1.36, 1.88) | <0.001 |
| Ethnicity |  |  |  |  |
| White | Ref |  | Ref |  |
| Black or African American | 1.31 (1.11, 1.55) | 0.002 | 1.36 (1.14, 1.63) | 0.001 |
| Asian | 0.61 (0.34, 1.12) | 0.11 | 0.72 (0.39, 1.33) | 0.30 |
| Native Hawaiian or other Pacific Islander | 1.96 (0.39, 9.75) | 0.41 | 2.35 (0.46, 12.0) | 0.30 |
| American Indian or Alaska Native | - | - | - | - |
| Unknown | 0.61 (0.46, 0.80) | <0.001 | 0.74 (0.56, 0.97) | 0.03 |
| Co-morbidities within the Charlson co-morbidity index | |  |  |  |
| Myocardial Infarction | 2.61 (2.15, 3.17) | <0.001 | 1.84 (1.48, 2.30) | <0.001 |
| Congestive Heart Failure | 2.15 (1.82, 2.54) | <0.001 | 1.34 (1.10, 1.64) | 0.004 |
| Peripheral Vascular Disease | 1.39 (1.15, 1.69) | 0.001 | 0.83 (0.67, 1.03) | 0.10 |
| Cerebrovascular Disease | 1.48 (1.24, 1.78) | <0.001 | 1.03 (0.84, 1.25) | 0.81 |
| Dementia | 1.71 (1.41, 2.05) | <0.001 | 1.42 (1.16, 1.74) | 0.001 |
| Chronic Pulmonary Disease | 1.45 (1.22, 1.71) | <0.001 | 1.12 (0.93, 1.35) | 0.23 |
| Rheumatic Disease | 1.25 (0.84, 1.87) | 0.27 | - | - |
| Peptic Ulcer Disease | 1.16 (0.73, 1.82) | 0.53 | - | - |
| Mild Liver Disease | 1.06 (0.74, 1.52) | <0.001 | 0.84 (0.57, 1.22) | 0.35 |
| Moderate/Severe Liver Disease | 0.39 (0.05, 2.93) | 0.36 | - | - |
| Diabetes without chronic complications | 1.41 (1.20, 1.67) | <0.001 | 0.98 (0.82, 1.18) | 0.86 |
| Hemiplegia or Paraplegia | 0.75 (0.45, 1.23) | 0.25 | - | - |
| Renal Disease | 2.50 (2.13, 2.93) | <0.001 | 1.88 (1.57, 2.25) | <0.001 |
| Any Malignancy | 1.15 (0.93, 1.41) | 0.93 | - | - |
| Metastatic Solid Tumour | 1.52 (1.01, 2.29) | 0.04 | 1.29 (0.85, 1.98) | 0.23 |
| AIDS/HIV | 0.97 (0.28, 3.29) | 0.96 | - | - |

CI: confidence interval, OR: Odds Ratio. American Indian or Alaska Native omitted because there were no deaths among this group. Only characteristics p<0.05 in the unadjusted analyses were included in the multivariate analysis.
