# Supplementary material for: Comorbidities associated with mortality in 31,461 adults with COVID-19 in the United States: A federated electronic medical record analysis
Source: PLoS Med. 2020 Sep 10;17(9):e1003321. doi: 10.1371/journal.pmed.1003321 (PMC7482833; doi:10.1371/journal.pmed.1003321)
Supplement: S1 Text — (DOCX) [file pmed.1003321.s005.docx]

TriNetX standardizes data, which have been extracted from EMR systems, in two ways:

1. Standardizing the structure, i.e., putting data into a common structure or data model.

TriNetX data providers deliver data in any of a variety of formats: Informatics for Integrating Biology and the Bedside (i2b2), Observational Medical Outcomes Partnership (OMOP), TriNetX ingestion file specification, the North American Association of Central Caner Registries (NAACCR) tumour registry structure, and others. TriNetX have product capabilities that have been tested extensively that map data from each of these structures to the common model within TriNetX.

2. Standardizing the terminology, i.e., mapping codes, units, etc, to a consistent standard

TriNetX have a team of Informaticists that map data from the data provider’s local codes to master terminology within TriNetX. For example, TriNetX map the provider’s medication codes to RxNorm medication codes. TriNetX map the provider’s local laboratory codes to LOINC laboratory codes.

To quality check that mapping, TriNetX review the mapping with the data provider, and TriNetX run data quality tools to identify anomalies and outliers that may indicate mappings that need to be changed. In the event that identify issues are identified within the mapping, the mapping is fixed.

There are occasions when data providers provide data that shows anomalies (e.g., an unusually high lab value, two encounters with the same encounter ID). In other words, these are issues not with our standardization of the data by TriNetX, but with the source data itself. In general, TriNetX hesitate to change source data values because changing the data would impose one interpretation of the data unilaterally. TriNetX prefer to give the data providers flexibility to interpret the data based on their own use case.

When TriNetX find any anomalies, the data providers are notified so that they can determine whether they have introduced an issue in their own data processing vs. whether the source data are consistent with what they provided to TriNetX.
